# Study of mechanical and electrical properties through positron annihilation spectroscopy for ethylene-propylene-diene rubber biocomposites with treated wheat husk fibers

**DOI:** 10.1038/s41598-024-73594-3

**Published:** 2024-10-16

**Authors:** Hamdy F. M. Mohamed, Howayda G. Taha, Wael M. Mohammed, Esam E. Abdel-Hady, Somia Awad

**Affiliations:** 1 Physics Department, Faculty of Science, Minia University, P.O. Box 61519, Minia, Egypt; 2 Radiation Safety Department, Nuclear and Radiological Safety Research Center, Atomic Energy Authority, Cairo, Egypt

**Keywords:** Ethylene-propylene-diene monomer rubber (EPDM), Wheat husk fibers (WHFs), Swelling, Positron annihilation lifetime (PAL), Positronium, Free volume, Electrical conductivity, Materials science, Nanoscience and technology, Physics

## Abstract

The positron annihilation lifetime (PAL) spectroscopy characteristics of ethylene-propylene-diene monomer rubber (EPDM) composites reinforced with treated wheat husk fibers (WHFs) were investigated for the first time. PAL spectroscopy is employed to study the free volume of polymers. The use of lignocellulosic materials as reinforcement in polymeric composites has gained attention due to their low cost, availability, and eco-friendliness. In this study, the impact of the loading concentration on the interfacial adhesion between the EPDM matrix and WHFs is quantified, along with the evaluation of swelling measurement and tensile properties. Additionally, the nanoscopic properties derived from PAL spectroscopy correlate with the composites’ macroscopic properties. In addition, the dielectric properties of the investigated samples have been studied, and their conductivity has been calculated. To determine the conduction mechanism within these samples and how it is affected by the addition of WHF, the change in electrical conductivity with the frequency of the external electric field applied to the samples was studied, and from this, the conduction mechanism was determined, and the barrier height value was calculated. The experimental results provide insights into the relationship between the structure and properties of EPDM-WHF biocomposites, offering valuable knowledge for developing sustainable and high-performance materials.

## Introduction

Positron annihilation lifetime spectroscopy (PALS) provides a new understanding of material characteristics in condensed matter^1^. Over the past few decades, this spectroscopy has proven to be the most effective experimental technique for examining the free volume in polymers. In this method, we use the bound (e^−^ & e^+^) pair, positronium (Ps), similar to hydrogen, as a probe for sub-nano-sized local free volumes in its stable orthostats, *o*-Ps^2^. Ps is trapped in a hole within the free volume of amorphous polymers, and the size of the hole it annihilates determines how long it will last^3–5^. Positronium (Ps) atoms can be formed, or positrons can be directly annihilated by electrons (the source of τ_2_) because of their interaction with polymeric materials^6,7^. Ps can occur in two states: *ortho*-positronium (*o*-Ps), where the two particles’ spins are parallel, and *para*-positronium (*p*-Ps), an electron-positron state with anti-parallel spins. With the shortest lifetime (τ_1_), *p*-Ps self-annihilate, whereas *o*-Ps are annihilated by a pick-off mechanism. The free volume size is determined by measuring the annihilation lifetime of positrons implanted in a polymer matrix and preferentially localized in vacancies, pre-existing defects, or voids (> 1 nm), or free volume (0.1–1 nm)^8–14^. On the other hand, a longer lifetime is linked to a larger size, whereas a shorter lifetime denotes a smaller size^15^.

Ethylene-propylene-diene monomer rubber (EPDM) has high tension, high tensile strength, toughness, and age resistance, which make it ideal for usage in a variety of fields^16,17^. Polymer composites have been created by combining several types of synthetic reinforcing filler with polymers to enhance their mechanical properties and achieve the desired features for real-world applications. Research is underway to determine how to replace synthetic fibres with lignocellulose fibre as reinforcing filler^18^. The use of lignocellulosic materials as reinforcement in polymeric composites has been the subject of more research in recent years since natural fibres can enhance the product by contributing attributes like low density and biodegradability. Furthermore, these materials come from less expensive and sustainable sources. As a result, a lot of natural fibres have been utilized in cars, trucks, and train cars^19^. Agro-based renewable materials have drawn a lot of interest from the scientific community recently because of their affordability, portability, environmental friendliness, and increased awareness of environmental issues globally^20–27^. They are now being developed as a possible substitute for mineral fillers in a broad range of industrial uses. Due to their biodegradability and low cost, composites made with natural reinforcing fillers have the potential to reduce environmental pollution and play a significant part in resolving ecological issues that we may encounter in the future^23^. Even if materials made entirely of renewable resources are more environmentally beneficial, some applications cannot use these materials because they lack the necessary performance characteristics. The combination of natural fibres and synthetic polymers offers not only a great opportunity to utilize agro-based natural resources but also demonstrates the desired performance qualities from a more sustainable material that would otherwise pollute the environment if disposed of or burnt.

In contrast to synthetic fibres like talc, silica, glass, carbon, and others, lignocellulosic fibres from sources like corn, straw, wheat husk, abaca, rice husk, and grass are inexpensive, readily available, renewable, and light on equipment^27–31^. In addition, natural fibre fillers have advantages over mineral fillers since they don’t scratch surfaces, use less energy during production, and lower the density of finished goods. As a result, these composites have garnered a lot of interest and are playing a bigger role in the creation of numerous low-cost, lightweight, environmentally friendly composites. The composition, characteristics, and filler-matrix interaction of lignocellulosic composites greatly influence their mechanical and physical attributes. A good compounding procedure and efficient component mixing will result in better filler dispersion. Through chemical and physical alteration of the filler or the use of appropriate coupling agents, compatibility between the two components can be achieved. When the composites are being prepared, coupling agents have the ability to react with both components, forming a chemical bridge at the filler-matrix interface^28,32^.

The primary drawbacks of utilizing lignocellulosic fibres as fillers in thermoplastics are their inadequate filler-matrix interaction, a strong propensity to absorb moisture, and ensuing alteration in the dimensions of the final product. If the matrix completely encases the filler and there is a positive filler-matrix interaction, the composite’s propensity to absorb moisture can be significantly decreased^28^. Numerous efforts have been undertaken to enhance filler-matrix interface bonding. Acetylation of the cellulose hydroxyl group in the fibre can significantly reduce its hydrophilic properties, according to Rowell et al.^33^. It is well known that wheat husk fibres, or WHFs, are an agricultural waste with the potential to be a substantial, reasonably priced, and ecologically benign filler derived from a renewable source.

Consequently, WHFs have a fascinating source that is being thoroughly studied. Where no great effort has been made thus far, recent progress should provide a range of commodities. To produce composites with the desired properties, the interface between the lignocellulosic filler material and the polymeric matrix must be improved. It is possible to promote interfacial adhesion in a system, including lignocellulosic materials, in several ways. To the best of our knowledge, no previous work was done about this system using the PAL spectroscopy. The objective of this research is to measure the effect of fibre loading on the interfacial adhesion between the wheat husk fibre (WHF) matrix and ethylene-propylene-diene monomer rubber (EPDM). Evaluations are also conducted on wide-angle x-ray diffraction (XRD), toluene swelling, composite tensile characteristics, and electrical conductivity capabilities. Furthermore, the free volumes of the nanostructure composites have been determined, and a successful correlation has been established between the macroscopic properties and the nanoscopic properties obtained using positron annihilation lifetime spectroscopy.

## Experimental

### Materials

Wheat husks were harvested from Upper Egypt’s Beni-Suef region. To eliminate any remaining unwanted dirt, clay, or dust, the wheat husks were thoroughly cleaned many times with tap water before being dried for an entire night at 102 ± 2 °C. After being dried and processed mechanically in a rotary cutting mill at 10,000 rpm to a size of 2 to 3.5 mm, the husks were automatically sieved into length-based categories. The fibre length that was examined was medium (125–250 μm). Table 1 shows the chemical composite of the wheat husk fibres. Uniroyal Chemical Co., Inc. of Naugatuck, USA, provided the ethylene-propylene-diene monomer (EPDM) rubber. Between 0.87 and 0.89 g/cm^3^, it had a specific gravity, and its price-earnings ratio (E/P ratio) was 57/43^43^. Other typical materials used in rubber compounding, like stearic acid, zinc oxide, and sulphur, are supplied by Transport and Engineering Company, TRANCO, Alexandria, Egypt. These ingredients are of commercial grade and don’t require any additional purification. Paraffin wax was utilized, which has a broad melting point of 35–75 °C and a molecular weight of 340–430 g/mol. The organic solvent used to measure swelling was toluene, which has a density of 0.866 gm/cm^3^.

**Table 1:** Chemical composition of wheat husk fibers (WHFs).

Composition	%
Lignin	16.4
Cellulose	30.5
Hemicellulose	28.9
Ash content	11.2–6.6
Others	2.4

### Sample preparation

The recipe given in Table 2 describes the mixes’ compositions. According to ASTM D3184–89 from the American Society for Testing and Materials, the composite materials were in a two-roll laboratory mill (150 mm × 300 mm) kept at almost 50 °C. For every combination, the mill/roll speed ratio, nip gap, and number of passes remained constant. At a 1.25 mm mill opening, the samples were ground for a long enough time to distribute the fibers throughout the matrix. After mixing, the WHF was added, making sure to consider the compound’s flow direction so that most of the fibers were pointing in the same direction.

**Table 2:** Base formulation of EPDM-WHF- composites.

Ingredients (phr)	EW0	EW30	EW40	EW50
EPDM	100	100	100	100
Zinc Oxide	5	5	5	5
Stearic acid	1	1	1	1
HAF^a^ (N-330)	30	30	30	30
Paraffin	5	5	5	5
WHF^b^	0	30	40	50
MBTS^c^	1	1	1	1
PbN^d^	1	1	1	1
Resin	5	5	5	5
Sulfur	2	2	2	2
Length, µm	125–250			

^a^High abrasion furnace black.

^b^Wheat husk fibers.

^c^Dibenthiazyl disulfide.

^d^Phenyl-β-naphthylamine.

### Characterization

In accordance with ASTM D1646 and D2084, rubber mixes were conditioned for 24 h at almost 25 °C before being evaluated for cure using a Monsanto ODR-100 Rheometer. The compounds were cured according to the obtained respective minimum torque *M*_*L*_, maximum torque *M*_*H*_, and cure time *T*_*C90*_^34^. The vulcanization process for the EPDM compounds was carried out in a hydraulic press at 150 °C and 4 MPa of pressure, in compliance with the optimal cure period ascertained by rheometer data. A uniform circular cut with a diameter of 5 mm and a thickness of 2 mm was used for the ASTM D471 swelling test, which was conducted using the immersion/gain method^43^ on compression-molded rubber samples. The samples were kept at room temperature for two days to enable the swelling to reach diffusion equilibrium. Tensile specimens were cut from 2 mm vulcanized composite sheets according to the longitudinal-transverse fibre orientations using a dumbbell-shaped cutter. Tensile tests (ASTM D412-98a) were conducted using a Zwick 1425 tensile testing apparatus at a crosshead speed of 50 mm/min. The tensile strength and ultimate elongation of the samples under investigation were tested at room temperature^35^.

A traditional fast-fast coincidence spectrometer with a time resolution of 280 ps was used for positron annihilation lifetime spectroscopy (PALS). It was done at high vacuum (5105 Torr) and at room temperature. A 20 µCi ^22^Na positron source was used, supported on a 7 μm-thick Kapton foil. To guarantee that the positrons annihilate in the sample at a thickness of approximately 1 mm, the foil is then sandwiched between 1 cm x1 cm of the samples. Approximately 2 million counts were gathered during an unusual 3-hour acquisition period for each sample. The LT programme^36^ was used to analyse the positron lifetime spectrum into three components without source correction with a variance ratio less than 1.2.

An X-ray diffractometer (GBC EMMA X-ray diffractometer, Australia) equipped with Cu Kα (= 1.54184 Å) radiation at 30 kV and 30 mA was used to examine the structure and crystallinity of wheat husk fiber rubber composites. A scintillation one-dimension position-sensitive detector was used to capture scattering profiles at a rate of 0.5º/min throughout a range of 5º < *2θ* < 80º, with a step of 0.05º. The measurements were done in the Central Laboratory of Microanalysis and Nanotechnology, Minia University.

Numerous parameters, including conductance, loss coefficient, parallel-equivalent static capacitance, and impedance, were measured for each sample using an LCR metre (Hioki 3532, Japan). The dielectric properties and complex impedance were computed as a function of frequencies from 50 Hz to 5 MHz. At 25 °C, the measurements were conducted in a vacuum. The out-of-plane value of the ac ionic conductivity σ_ac_ was calculated using the conductance *G* as^37^;

1 $$\it {\sigma _{ac}}=\frac{\text{L G}}{\text{A}}$$

where *L* and *A* is the film thickness and the cross-sectional region of the electrodes, respectively.

## Results and discussion

### Wide angle X-ray diffraction

**Fig. 1:**
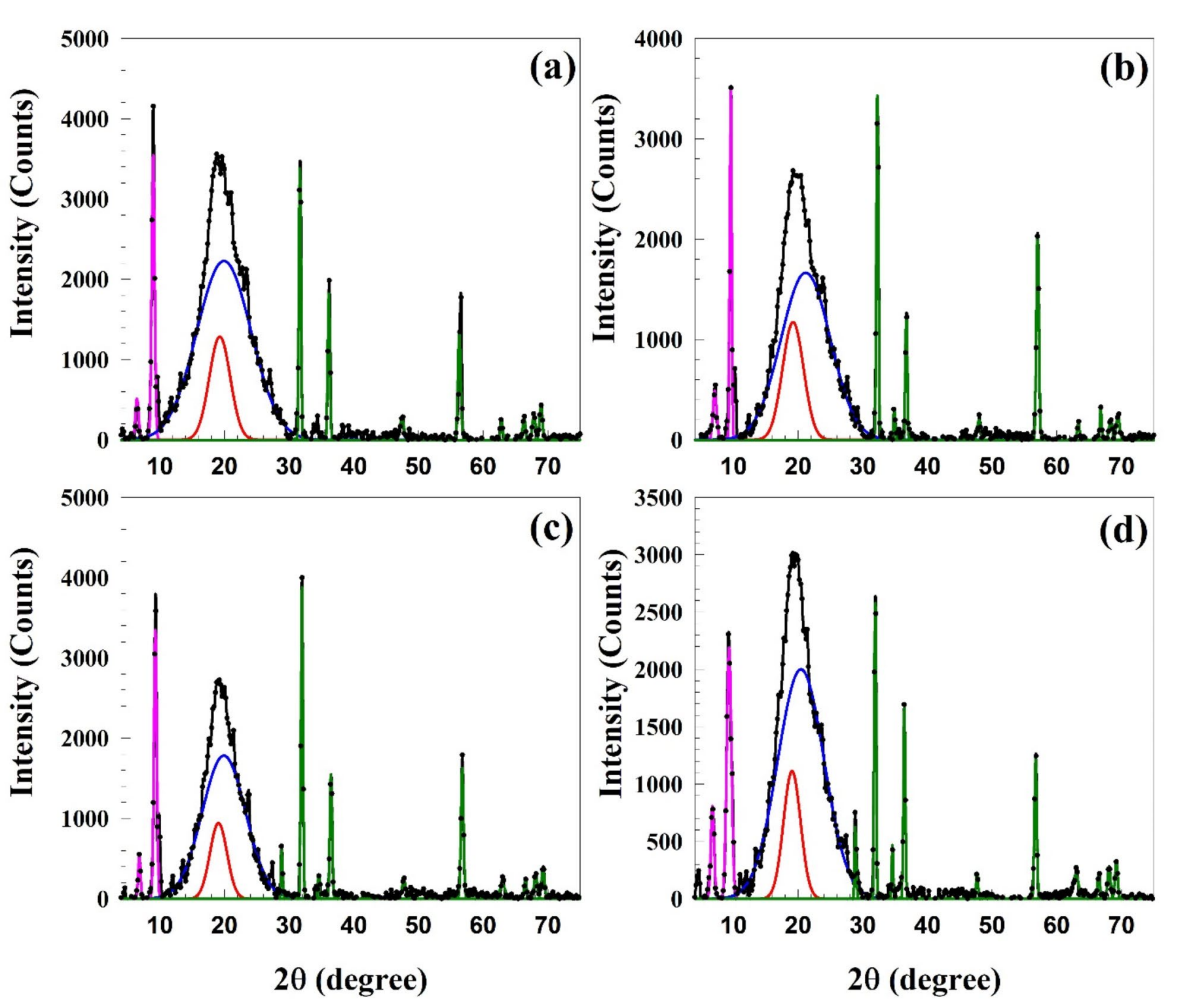
Wide angle x-ray diffraction patterns for EPDM with different concentrations of WHF in EPDM (a) EW0, (b) EW30, (c) EW40, and (d) EW50. Red colour peak represents the crystalline peak while blue peak represents the amorphous peak.

Wide angle X-ray diffraction (WARD) patterns of ethylene propylene diene monomer (EPDM) with different concentrations of wheat husk fibers (WHF) were used to investigate the influences of WHF on the structural performance of nanocomposites, as shown in Fig. 1. The WAXD pattern of the samples involved a strong peak at *2θ* around 10^o^ which connected the significant peaks for high abrasion furnace black (HAF, N-330) of the carbon atom^38^ for the plane (002). The broad peak with a center at *2θ* around 18^o^ could be connected to the semicrystalline structure of EPDM polymers^39^. Moreover, the three strongest peaks at *2θ*  = 32^o^ (100), 35^o^ (002), 56^o^ (110), and the many lower intensity peaks at 63^o^ (103), 68^o^ (112), 69^o^ (201), are corresponding to the zinc oxide (ZnO) nanoparticles^38,40^. Using the WAXD pattern and the aid of the Fityk programme, the samples’ degree of crystallinity were calculated^41^. The amorphous and the crystalline peaks, represented by red and blue coloured, respectively [Fig. 1], were separated from the pattern’s mean peak, which ranged from 10 to 30 degrees. The sample’s amorphous portion is indicated by the wider peak, whereas the sample’s crystalline portion is revealed by the sharper peak. It is possible to calculate the degree of crystallinity *X*_c_ as follows:

2 $${X_c}\left( \% \right)=\frac{{Area\;~under\;~the\;~sharper~\;sup~\;peak}}{{Total~\;area\;~under\;~the\;~main\;~peak}}~ \times 100$$

The Scherrer equation^42^ was utilized to calculate the grain size D, which is equal to

3 $$D=\frac{{0.9\lambda }}{{\beta {\text{cos}}\left( \theta \right)}},$$

where λ is the wavelength, and β is the full width at half maximum (FWHM). The FWHM, the grain size, and the peak position for both crystalline and amorphous peaks are listed in Table 3. Table 3 also includes a list of the prepared samples’ degree of crystallinity. It is clear from the table that the degree of crystallinity increases with increasing the WHF concentration up to 30 phr and then decreases with increasing the concentration of WHF from 30 to 50 phr in the composite. This is due to the fact that, as the concentration of WHF rises, the chain entanglement also rises, as shown by the larger grain sizes of the crystalline and amorphous components. The data of Sobhy and Tammam^43^, who discovered that the crosslinking density increased with an increase in the WHF concentration in the compound, were supported by the current results. Later on, as we will explore, a decrease in the degree of crystallinity and an increase in the free volume resulted from the creation of a crosslinking network and the shrinkage of the dangling segment^44^.

**Table 3:** The peak position, FWHM, and grain size for the crystalline and amorphous peaks for the EPDM polymers with different concentrations of WHF.

WHF concentration (phr)	Crystalline peak			Amorphous peak			Degree of crystallinity (%)
	Peak position (degree)	FWHM (degree)	Grain size (nm)	Peak position (degree)	FWHM (degree)	Grain size (nm)	
0	19.26	3.82	2.20	19.91	9.88	0.85	18.20 ± 0.91
30	19.20	3.84	2.19	21.16	8.97	0.94	23.20 ± 1.18
40	19.06	3.11	2.71	19.90	7.89	1.07	17.24 ± 0.86
50	19.05	3.11	2.71	20.42	8.42	1.00	17.05 ± 0.84

### Vulcanization behaviour

**Fig. 2:**
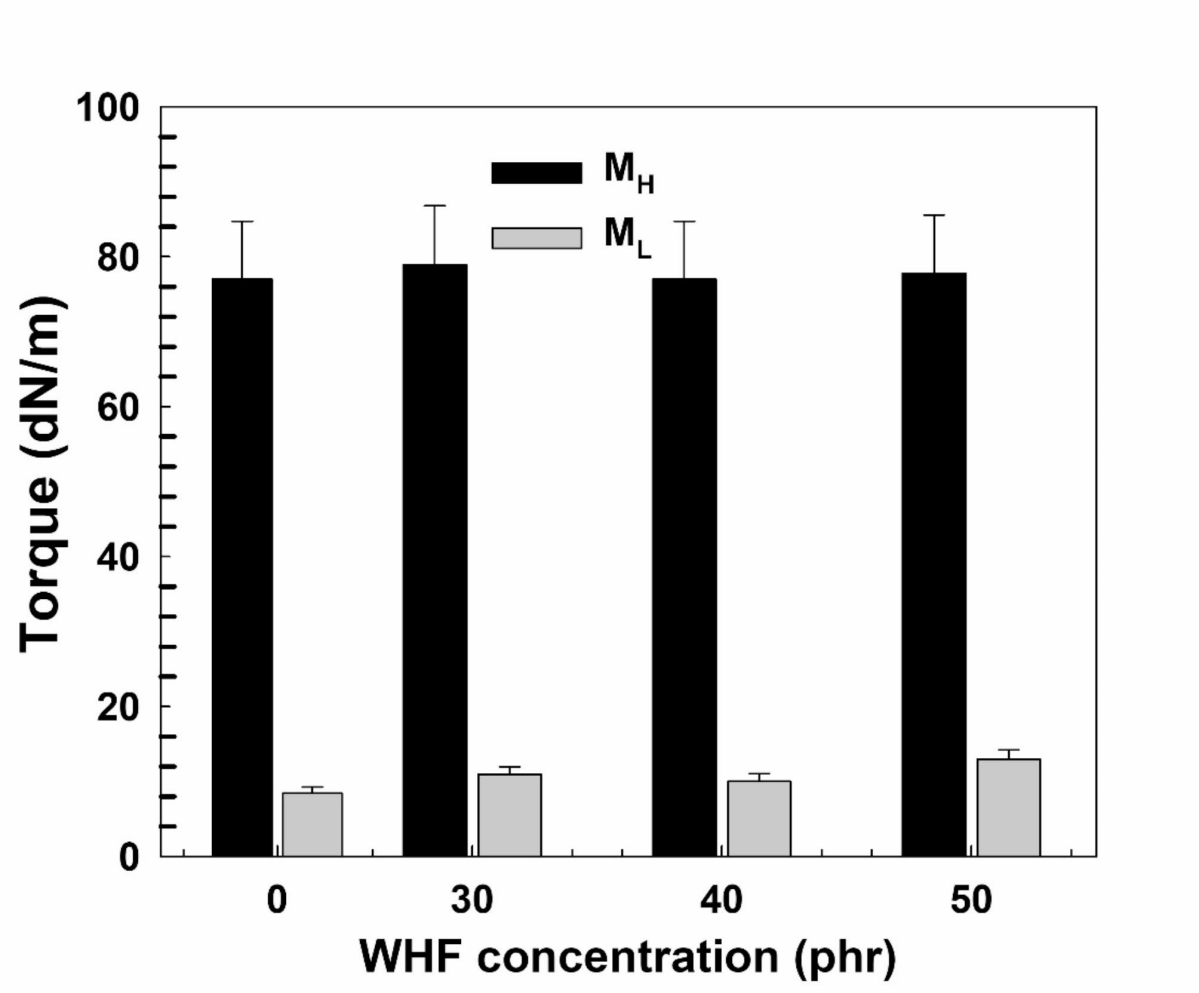
Maximum torque M_H_ and lowest torque M_L_ variation with WHF concentration in EPDM.

It is commonly recognised that the presence of fibres or rubber crosslinking causes an increase in torque, and that the torque levelling off signifies the end of the curing process. Figure 2 shows data on the cure characteristics at various WHF concentrations. The increase in the torque values from the minimum *M*_*L*_ to the maximum *M*_*H*_ indicates the increase in the stiffness of the fiber-reinforced materials. The cure time *T*_*C90*_ values are determined as 19.70, 20.75, 18.83, and 20.30 min for EW0, EW30, EW40, and EW50, respectively. The present results seemed to be dependent mainly on the rubber matrix where the increase in fiber concentration in EPDM mixes does not have a considerable effect on the cure time, that is, the rubber phase plays a crucial role in the performance of based natural fiber polymer composites^45^.

### Influence of fibre content on the mechanical properties

**Fig. 3:**
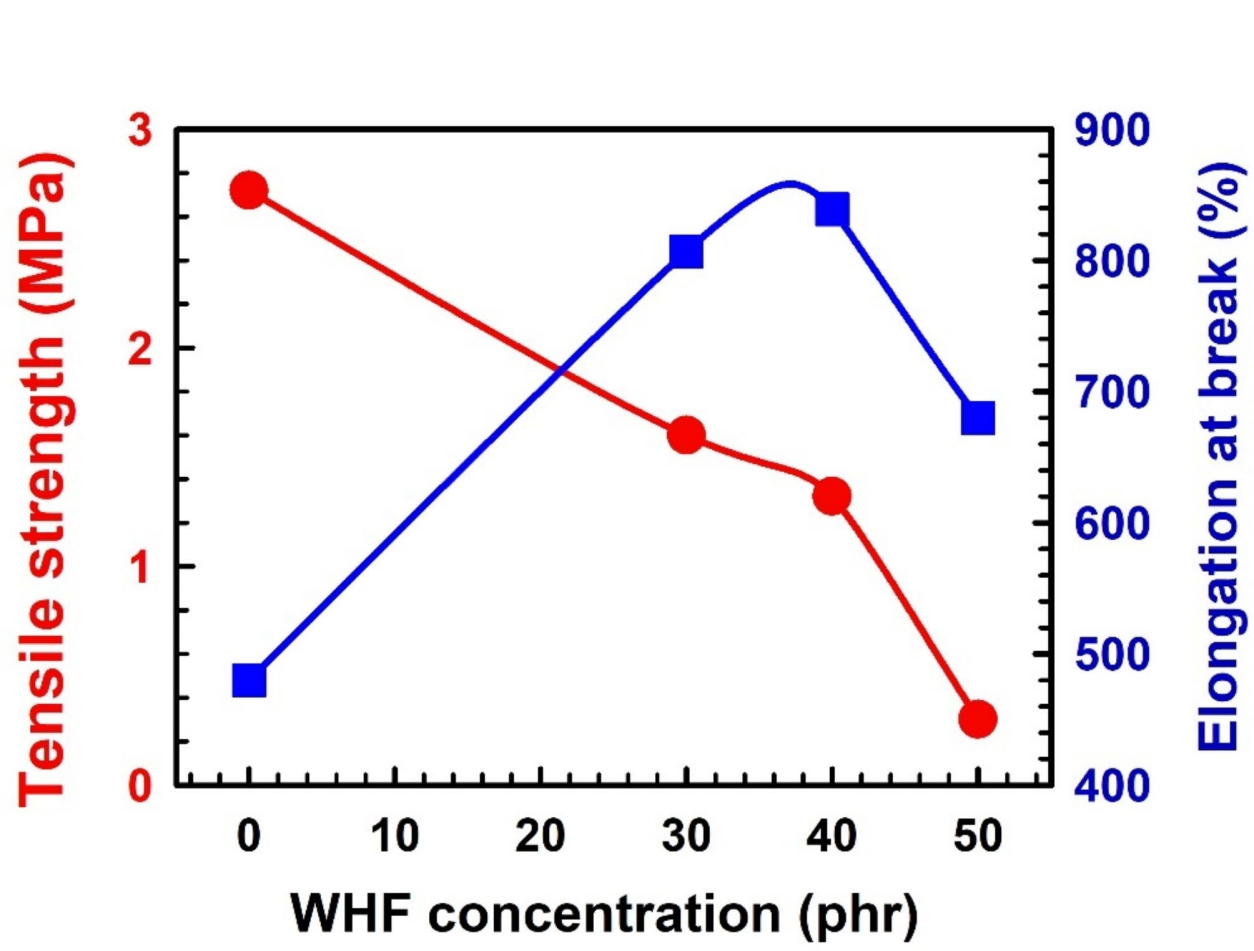
WHF concentration in EPDM dependence of the tensile strength [●] and elongation at break [■]. The error bars are within the symbol’s sizes.

Figure 3 illustrates the tensile strength behaviour of medium length composites with 0, 30, 40, and 50 phr of WHF in EPDM. The results show that when the filler fraction increases, the tensile strength drops. Other researchers have discovered similar results^46,47^. It implies that when the fibre content rises, WHFs have a tendency to stick together in bundles and inhibit the dispersion of the individual fibres. These demonstrate that the poor tensile strength up to 30 phr is primarily due to the fibers and rubber matrix not adhering to one another. However, there are enough fibers to hold the matrix in place, and the fibers start to strengthen the matrix after 40 phr, when the stress distribution is homogenous. Similar patterns were mentioned by Murty and De^48^, Setua and De^49^, and Dzyura^50^. Figure 3 also shows that EPDM composites’ elongation at break increases in tandem with WHF loading, but only up to a concentration of 40 phr. It indicates that when filler loading increases, elongation at break decreases as filler loading increases, indicating a greater limitation on the macromolecules’ ability to move molecularly. WHF tends to increase flow resistance as a result, decreasing resistance to break^51^. The results demonstrated that the fibre has a greater stiffening impact than an unfilled one since elongation is proportional to a material’s rigidity^52,53^.

### Effect of fibre loading on measurements of swelling

**Fig. 4:**
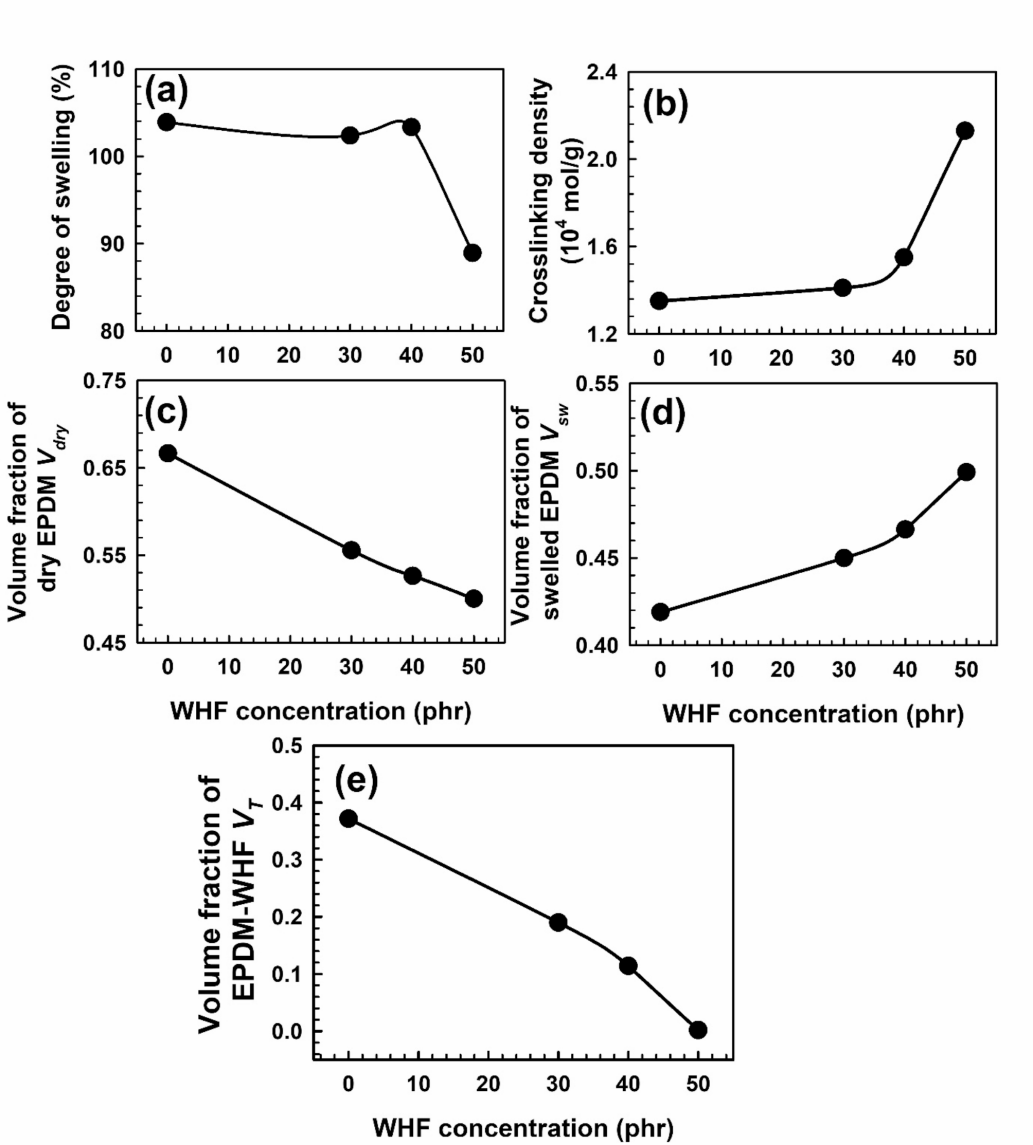
The WHF concentration dependence of the volume fraction of (a) degree of toluene swelling, (b) crosslinking density, (c) EPDM in dry *V*_*dry*_, (d) EPDM in swollen with toluene *V*_*sw*_, and (e) EPDM-WHF mixtures *V*_*T*_ swelling in toluene. The error bars are within the symbol’s sizes.

Many articles have addressed the swelling characteristics of filled rubber vulcanizates in various solvents^54,55^. The quantity of swelling was managed at equilibrium using the Flory-Rehner hypothesis^56^ for rubbery polymer swelling:

4 $$\ln \left( {1 - {V_r}} \right)+{V_r}+\chi V_{r}^{2}+{V_c}{V_1}\left( {V_{r}^{{\frac{1}{3}}} - \frac{{{V_r}}}{2}} \right){V_0}=0,$$

where *V*_*c*_ is the number of “moles” of the effective network chain in the initial or unswollen volume *V*_*0*_, *V*_*1*_ is the molar volume of the solvent, χ is the polymer-solvent interaction parameter, and *V*_*r*_ is the volume fraction of rubber after swelling. Figure 4a, b shows the relation between the WHF loading and both the degree of swelling of toluene and crosslinking density. WHF concentration increases cause the compound’s crosslinking density values to rise and its absorption solvent values to drop. The reason behind this is that when the WHF rises, the chain entanglement also rises, which lowers the solvent uptake.

The degree of constraint imposed by the reinforcing filler could be understand using the equation of Kraus^54,55,57^:

5 $$\frac{{{V_{ro}}}}{{{V_{rf}}}}=1 - \frac{{\left[ {3c\left( {1 - {V_{ro}}} \right)+{V_{ro}} - 1} \right]}}{{\left[ {\frac{\phi }{{1 - \phi }}} \right]}}.$$

In this instance, *V*_*ro*_ and *V*_*rf*_ are the volume fractions of rubber in the filled and unfilled vulcanizates, respectively,* φ* represents the volume fraction of the filler, and c is the filler’s constant characteristic that indicates the degree of adhesion. The volume fraction of EPDM in the dry *V*_*dry*_, swollen *V*_*sw*_, and $\:{V}_{T}\:(={V}_{dry}-\frac{{V}_{sw}}{{V}_{dry}})$ EPDM-WHF mixtures is used to evaluate the interfacial adhesion between fiber and rubber, as shown in Fig. 4c-e. The *V*_*T*_ values of composites with greater WHF concentrations are significantly lower than those without filler. Das^58^ found that improved adhesion between the rubber and fibre decreased the factor *V*_*T*_ by more than 0.04 units. This suggests that the higher-loaded compound would be more resistant to swelling than the flat compound. As a result, the relative values of *V*_*sw*_ and *V*_*T*_ would be higher and lower, respectively. Conversely, low bonding will cause *V*_*sw*_ to be relatively low, which will raise the *V*_*T*_ number. The amount of liquid that penetrates at equilibrium swelling decreases as fiber loading rises. This is because fiber exerts more resistance with higher stress.

### Positron annihilation lifetime

Without a precise definition, the concept of free volume can be utilised to explain a variety of polymer properties^59^. For instance, mechanical properties and the polymer’s free volume percentage^60^ are usually correlated (negatively). The applied load’s propensity to concentrate in the free volume rather than spread among the molecules of the polymer could lead the material to fail. Consequently, better mechanical properties are usually exhibited by a polymer with a reduced free volume percentage^61^. The PAL spectra were deconvoluted into three lifetime components using the LT programme^36^. The non-linear fit is stabilized by fixing the shortest lifetime τ_1_ at the *para*-positronium (*p*-Ps) lifetime of 125 ps. It is determined that the *ortho*-positronium (*o*-Ps) pick-off process is responsible for the longest lifetime τ_3_. The intermediate lifetime τ_2_ of the sample is due to both trapping and direct annihilation. Table 4 shows the lifetime fitting findings as a function of WHF loading in EPDM. With relative intensities of *I*_*1*_ + *ΔI*_*1*_, *I*_*2*_ + *ΔI*_*2*_, and *I*_*3*_ + *ΔI*_*3*_, respectively, the primary lifetime components τ_1_ + Δτ_1_, τ_2_ + Δτ_2_, and τ_3_ + Δτ_3_, mirror the free-volume size and positron trapping site nature of the sample. The bulk positron lifetime τ_b_ overlaps the first component, τ_1_ + Δτ_1_, which is the shortest lifetime component, with relative intensity *I*_*1*_ + *ΔI*_*1*_ attributed to the *p*-Ps annihilation. The bulk positron lifetime τ_b_ can be determined using Eq. (6) and recorded in Table 4 when *I*_*3*_ is significant.

**Table 4:** Positron annihilation lifetime parameters.

WHF concentration (phr)	I_1_ (%)	I_2_ (%)	I_3_ (%)	τ_2_ (ns)	τ_3_ (ns)	τ_b_ (ns)
0	30.12 ± 0.80	58.69 ± 0.96	11.19 ± 0.50	0.353 ± 0.004	2.469 ± 0.051	0.243 ± 0.009
30	23.53 ± 0.45	66.39 ± 0.24	10.08 ± 0.16	0.410 ± 0.003	2.592 ± 0.110	0.283 ± 0.008
40	19.68 ± 0.49	69.30 ± 0.90	11.02 ± 0.28	0.340 ± 0.002	2.517 ± 0.088	0.273 ± 0.009
50	32.08 ± 0.41	54.74 ± 0.37	13.18 ± 0.30	0.410 ± 0.004	2.385 ± 0.083	0.253 ± 0.010

6 $$\frac{1}{{{\tau _b}}}=\frac{{{I_1}}}{{{\tau _1}}}+\frac{{{I_2}}}{{{\tau _2}}}+\frac{{{I_3}}}{{{\tau _3}}}.$$

In accordance with the model presented by Shpotyuk et al.^62^ there are two possible types of defects in which positrons and Ps could become trapped where the symbols *K*_*d1*_ and *K*_*d2*_ represent the trapping rates of positron in the defects and the trapping rate of Ps in the free volume, respectively, as.

7 $${K_{d1}}={I_2}\left( {\frac{1}{{{\tau _1}}} - \frac{1}{{{\tau _2}}}} \right),$$

8 $${K_{d2}}={I_3}\left( {\frac{1}{{{\tau _1}}} - \frac{1}{{{\tau _3}}}} \right).$$

**Fig. 5:**
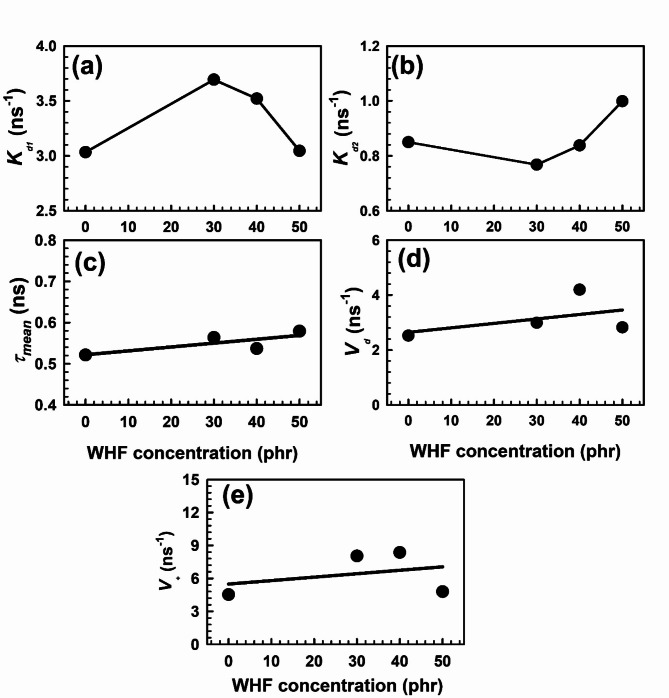
Variation of (a) *K*_*d1*_, (b) *K*_*d2*_, (c) τ_mean,_ (d) *V*_*d*_, and (e) *V*_*+*_ with WHF concentration. The error bars are within the symbol’s sizes.

EPDM is a semicrystalline polymer; hence, *K*_*d1*_ can be attributed to the positron annihilation rate in crystalline area defects and *K*_*d2*_ to the Ps annihilation rate in the free volume holes. Figure 5a, b shows the values of *K*_*d1*_ and *K*_*d2*_ as functions of the WHF loading. Both *K*_*d1*_ and *K*_*d2*_ exhibit the opposite behavior. Because EPDM is semicrystalline, as can be seen in the figure, the amorphous region traps less light than the crystalline region. X-ray studies also confirm that increasing the crosslink density with increasing WHF concentration will cause chains to entangle, increasing the number of free volumes more than the size of free volume.

The average positron lifetime *τ*_*mean*_ determined by analysing the PALS spectra’s results, is provided by;

9 $${\tau _{mean}}=\frac{{{\tau _1}{I_1}+{\tau _2}{I_2}+{\tau _3}{I_3}}}{{{I_1}+{I_2}+{I_3}}}.$$

Figure 5c shows the results of the τ_mean_ and it is evident that as WHF concentration rises, the τ_mean_ increases. The mechanical data support the hypothesis that an increase in crosslink density with increased WHF accounts for this behaviour. Assuming that “free positron annihilation rate” refers to annihilation of positrons in regions other than the open-volume defects, *o*-Ps trapping rate estimations in polymer matrix disordered regions were calculated using the bulk positron lifetime τ_b_ [Eq. (6)]. The bulk positron lifetime reciprocal is the same as the free positron annihilation rate. One may compute the Ps trapping rate *V*_*d*_ in the semicrystalline polymer’s amorphous regions using the formula^63^;

10 $${V_d}=\frac{{4{I_3}\left( {\frac{1}{{{\tau _b}}} - \frac{1}{{{\tau _3}}}} \right)}}{{3 - 4{I_3} - 3{I_2}}},$$

where the average bulk lifetime τ_b_ was found to be 0.263 ns across the whole range of WHF concentration in the EPDM polymer matrix. Figure 5d displays the calculated Ps trapping rate *V*_*d*_, and it appears that *V*_*d*_ grows linearly as the WHF concentration rises. This is caused by increasing the Ps trapping sites because of chain entanglement, which will increase the number of free volumes even though, logically, the average free volume size dropped with rising WHF. The trapping rate of positrons *V*_*+*_ in the vacancy type defect in the ordered or crystalline area of the EPDM polymer can be calculated as

11 $${V_+}=\frac{{3{I_2}\left( {\frac{1}{{{\tau _b}}} - \frac{1}{{{\tau _2}}}} \right)}}{{3{I_1} - {I_3}}}.$$

The *V*_*+*_ values in EPDM are displayed in Fig. 5e in relation to the WHF contents. The behavior of *V*_*+*_ as a function of WHF loading is observed to increase linearly with increasing the WHF contents up to 40 phr, then start to decrease. This is because of increasing crystallinity and crosslink density as WHF increases in the EPDM matrix. Moreover, *V*_*+*_ values were found to be greater than *V*_*d*_ values due to the decrease in free volume content.

If Ps is hosted in a spherical cavity with an effective radius of *R*, the *o*-Ps lifetime τ_3_ is proportional to the free volume hole radius *R* according to the Tao-Eldrup semi-empirical Eqs^64,65^;

12 $$\tau_{3}=0.5\left\{ {1 - \frac{R}{{Ro}}+\frac{1}{{2\pi }}\sin \left( {\frac{{2\pi R}}{{Ro}}} \right)} \right\} ^{-1}\;\;\left( {{\text{ns}}} \right),$$

where *R* and τ_3_ are expressed in the units of nm and ns, respectively, and *ΔR* = *R*- *R*_o_ is constant and found to be 0.1656 nm^66^. The calculated *R* from Eq. (12) was used to calculate the mean size of the free volume hole *F*_*V*_,

13 $${F_V}=\frac{4}{3}\pi {R^3}.$$

**Fig. 6:**
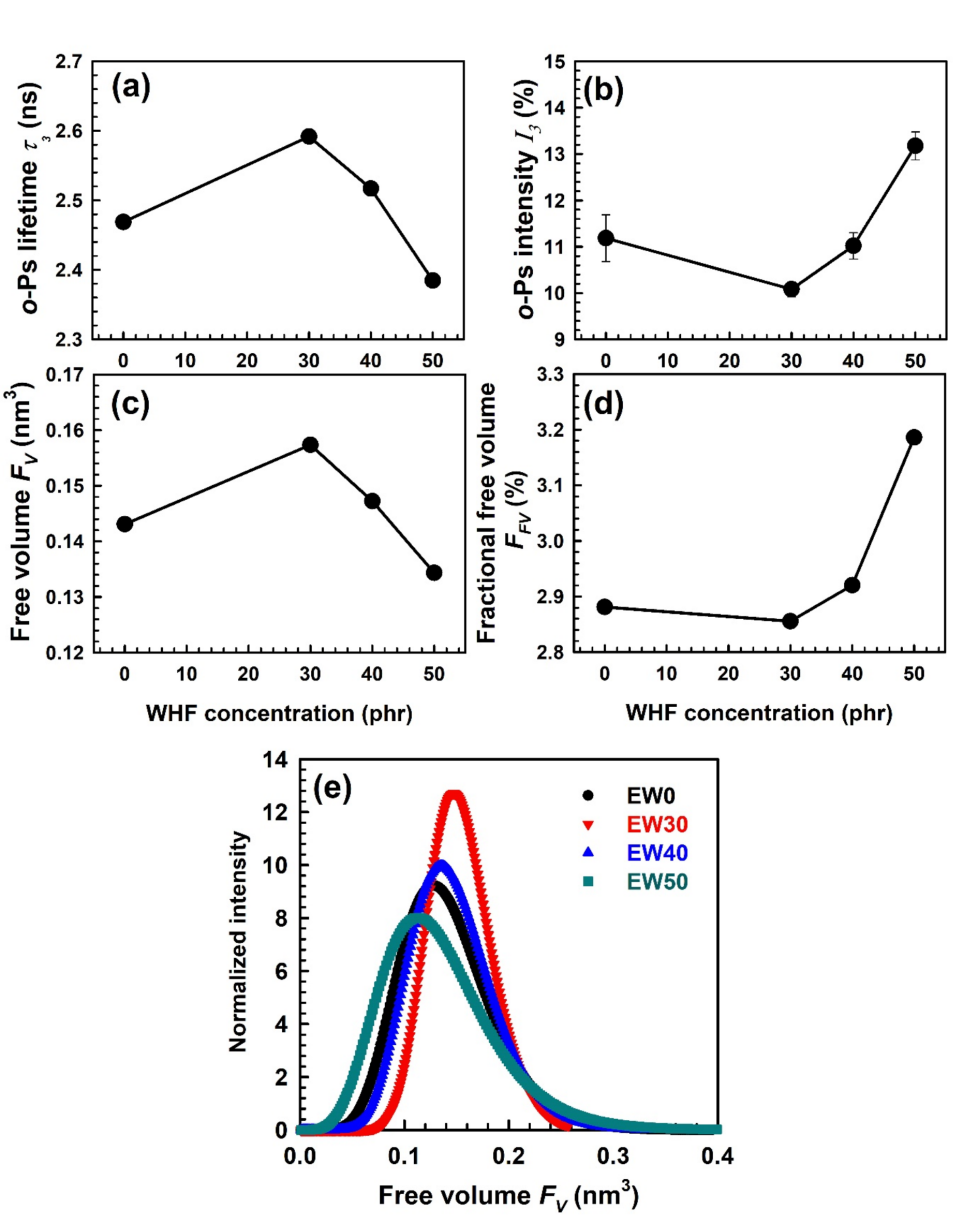
WHF concentration dependence of (a-d) positron annihilation parameters (τ_3_, *I*_*3*_, *FV*, and *FFV*) where the error bars are within the symbol’s sizes and (e) free volume distribution at different concentration of WHF in EPDM.

Using the *o*-Ps intensity *I*_*3*_ and *F*_*V*_, the fractional free volume *F*_*FV*_ is estimated according to the following equation:

14 $${F_{FV}}=C{\text{ }}{F_V}{I_3},$$

where C is empirically determined to be 1.8 nm^− 3^ from the specific volume data^67^. The *o*-Ps lifetime τ_3_, its intensity *I*_*3*_, free volume size *F*_*V*_, and fractional free volume *F*_*FV*_ of EPDM-WHF biocomposites as a function of WHF loading are shown in Fig. 6a-d. The *F*_*FV*_ has the same behaviour as *o*-Ps intensity *I*_*3*_, as was evident. Furthermore, the drop in τ_3_ indicates a reduction in the average size of polymer free volume holes, where EPDM composites with *F*_*FV*_ showed the long τ_3_ and small *F*_*FV*_ for a 30 phr concentration of WHF. The reason for this is that WHF prefers to establish hydrogen bonds at low loading levels (the huge surface area of WHF fibre helps), which restricts the mobility of EPDM chains and causes τ_3_ to increase.

Several physicochemical aspects and dynamical features are not entirely explained by analysing the mean free volume hole size and its concentration within the framework of the free volume theory. Alternatively, the free volume hole size distribution is the main issue in providing a succinct understanding of the free volume behaviour in the polymer matrix. The LT programme^36^ is used to find the distributions of the free volume hole in order to get more specific information on the free volume features. The LT software has proven to be efficacious in analysing the positron lifetime spectra of polymers^68^. The PAL spectrum exhibits a continuous decline, which can be represented mathematically. This model assumes that the true PAL spectra consist of a continuous distribution, rather than a discrete format^36^

15 $$y\left( t \right)=R\left( t \right)*\left[ {{N_t}\mathop \smallint \limits_{0}^{\infty } \left( {I\left( \tau \right)/\tau } \right)\exp ( - t/\tau )d\tau +B} \right]~$$

where

16 $$\mathop \smallint \limits_{0}^{\infty } I\left( \tau \right)d\tau =1$$

and *N*_*t*_ is the PAL spectrum’s total count. The continuous term analysis has been conducted using the standard LT10.0 programme^36^. For this analysis, the model function described in Eq. (15) is employed to fit the experimental data points, considering the number of terms in the spectrum. The LT10.0 programme, is utilized to determine the annihilation rates *λ* of positronium in free volume. These rates, defined as 1/τ_3_, are assumed to follow a normal distribution as^36^;

17 $${\alpha _3}\left( \lambda \right)= - {\left( {2\pi } \right)^{ - 0.5}}\sigma _{3}^{{ - 1}}exp\left\{ { - \frac{{{{\left[ {Ln\left( \lambda \right) - Ln\left( {{\lambda _{30}}} \right)} \right]}^2}}}{{2\sigma _{3}^{2}}}} \right\}{\lambda ^{ - 1}}d\lambda$$

where σ_3_ denotes the spread of the distribution, and *Ln* signifies the natural logarithm. Consequently, the *o*-Ps lifetime distribution α_3_ (τ_3_) is given by

18 $$\begin{aligned} {\alpha _3}\left( {{{\tau}}}_3 \right) & = - {\left( {2\pi } \right)^{ - 0.5}}\sigma _{3}^{{ - 1}}exp\left\{ { - \frac{{{{\left[ {Ln\left( \lambda \right) - Ln\left( {{\lambda _{30}}} \right)} \right]}^2}}}{{2\sigma _{3}^{2}}}} \right\}{\lambda ^{ - 1}}d\lambda\frac{{d{\tau_3}}}{{d\lambda }} \\ & = - {\left( {2\pi } \right)^{ - 0.5}}\sigma _{3}^{{ - 1}}exp\left\{ { - \frac{{{{\left[ {Ln\left( \lambda \right) - Ln\left( {{\lambda _{30}}} \right)} \right]}^2}}}{{2\sigma _{3}^{2}}}} \right\}{\lambda ^{ - 1}}d{\tau_3} \\ \end{aligned}$$

here *λ*_*30*_ is the peak annihilation rate of positronium in free volume. So that the free volume distribution was computed using Eqs. (12,13, 18) and is expressed as^12^;

19 $${\alpha _3}\left( V \right)= - {\left( {2\pi } \right)^{ - 0.5}}\sigma _{3}^{{ - 1}}exp\left\{ { - \frac{{{{\left[ {Ln\left( \lambda \right) - Ln\left( {{\lambda _{30}}} \right)} \right]}^2}}}{{2\sigma _{3}^{2}}}} \right\}{\lambda ^{ - 1}}d\lambda \frac{{dV}}{{d\lambda }}$$

where

20 $$\frac{{dV}}{{d\lambda }}= - \frac{{4\pi R_{v}^{2}{{\left( {{R_v}+{R_o}} \right)}^2}}}{{2{R_o}}}{\left\{ {1 - cos\left\langle {\frac{{2\pi {R_v}}}{{{R_v}+{R_o}}}} \right\rangle } \right\}^{ - 1}}$$

as derived from Eqs. (12 and 13). Figure 6e displays the free volume distributions for pure EPDM with varying WHF loading. In the following order, we may see a broader distribution in hole size: EPDM < EPDM-WHF, however, the free volume distribution for EPDM is broader than that for 30 phr WHF. Since the polymer chain’s motion can be reflected in the *o*-Ps lifetime distribution, the differences seen across all samples can be explained in terms of the packing of the polymer chain following the incorporation of WHF into the EPDM matrix. Moreover, the broader peak with increasing WHF means that the number of free volumes is increasing due to the rise in crosslink density and the subsequent chain entanglement. The peak of the distribution shifting to lower values may be due to two reasons. The first reason may be due to the decline in free volume content because of the increase in cross-link density. The second reason may be due to the motion of the polymer chain, and hence the difference manifest in all samples may be interpreted in terms of polymer chain packing after incorporation of WHF fillers, where embedding WHF into the EPDM matrix may restrict the polymer’s chain motion and result in decreasing the free volume hole size values.

### Dielectric properties study

**Fig. 7:**
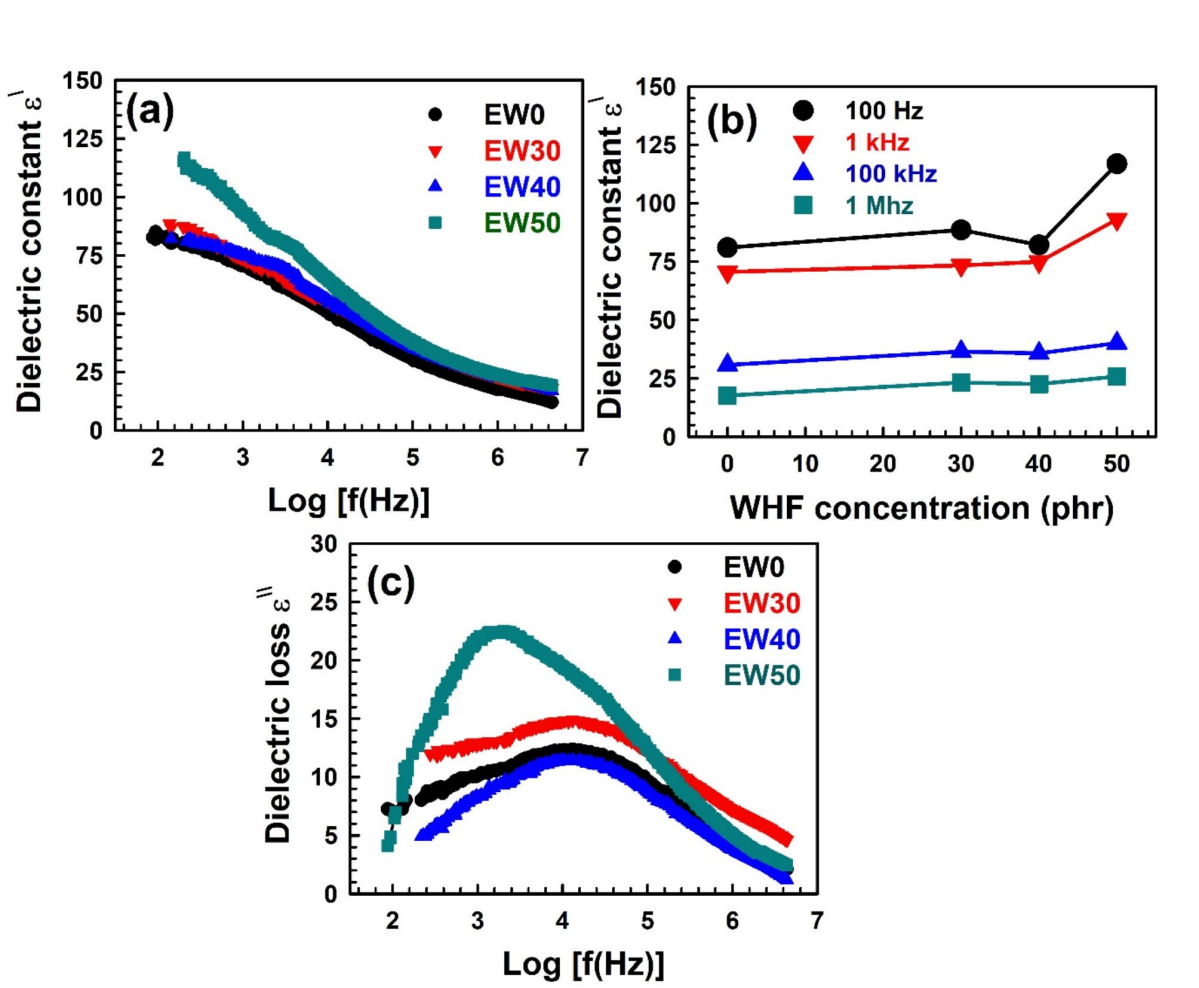
(a) Dielectric constant as a function of frequency for different WHF concentration in EPDM, (b) dielectric constant as a function of WHF concentration in EPDM at different frequencies, and (c) dielectric loss as a function of frequency for different concentration of WHF in EPDM.

As seen in Fig. 7a, the dielectric constant was generally found to decrease with frequency. Numerous investigations^42,69^ reported similar behaviour, which could be related to the involvement of many polarization components. The displacement of the valence electrons with respect to the positive nucleus is the initial sign of electronic polarization. Second, ionic polarization results from the displacement of positive and negative ions with respect to one another. Third, dipolar polarization results from molecules having persistent electrical dipole moments that can shift their orientation in the direction of an applied electric field. Finally, interfaces’ impedance mobile charge carriers cause space charge polarization. The sum of all these polarizations represents the total polarization of the material; the total polarization of any material is related to the dielectric permittivity^70,71^. The dielectric constant of WHF-EPDM samples shows high values at low frequencies; this is related to interfacial polarization which is the predominant type, and work on reducing the dielectric constant with increasing frequency, as was observed in a previous study^72^. By considering the effect of adding WHF, one can easily observe that increasing the WHF ratio has led to an increase in the samples’ ability to store electrical energy, as shown in Fig. 7b.

Figure 7c displays the dielectric loss as a function of frequency. The broad peak in the image displays dielectric relaxation behaviour. The free volume present in the samples is correlated with the dielectric relaxation in either pure EPDM or EPDM combined with varying amounts of WHF. Increased electrical conductivity and the reconstruction of electrically conductive pathways for hopping electrons are the causes of this^73^. Furthermore, as the WHF ratio increases, Fig. 7c demonstrates that the intensity under peaks has increased as well; this is consistent with the published study by Kuang et al.^74^. This makes sense because the WHF ratio, particularly at 50 phr of WHF, increases the free volume size. Debye relaxation theory can be used to explain how polar molecules behave in a dielectric material when subjected to an electric field. It explains a number of phenomena, including dielectric loss, which occurs when energy is lost as heat as a result of molecular friction during polarisation. It also clarifies why the dielectric constant of particular materials varies with frequency, indicating that the frequency at which the material can store electrical energy varies. One way to express the dielectric constant and dielectric loss is as follows:

21 $${\varepsilon ^\prime }={\varepsilon _\infty }+\frac{{{\varepsilon _s} - {\varepsilon _\infty }}}{{1+{\omega ^2}{\tau ^2}}},$$

22 $${\varepsilon ^{\prime \prime }}=\frac{{{\varepsilon _s} - {\varepsilon _\infty }}}{{1+{\omega ^2}{\tau ^2}}}\omega \tau +\frac{\sigma }{{\omega {\varepsilon _0}}}.$$

Equations (15) and (16) represent Debye relaxation theory, where and are the static and relative dielectric permittivity, respectively; is the dielectric constant in vacuum; is alternating conductivity; represents the relaxation time of polarized dipoles; and is the angular frequency. According to this hypothesis, the polar molecules in a dielectric material will align themselves with the electric field when it is applied. The material undergoes a shift in polarization throughout this alignment process, which takes some time. The material’s relaxation time dictates how quickly this polarization shifts^75,76^.

#### Electrical conductivity and conduction mechanism

**Fig. 8:**
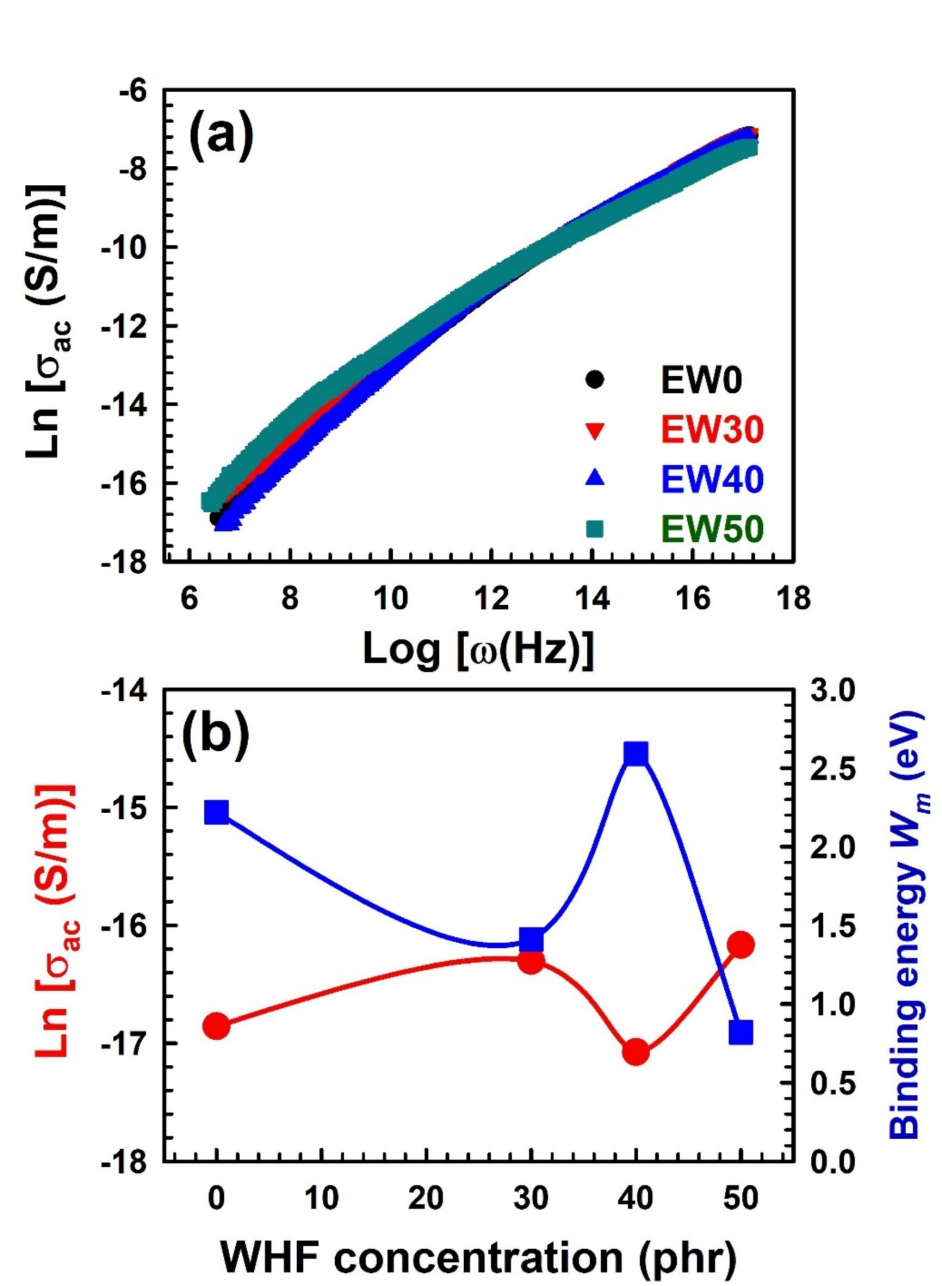
(a) The ac conductivity as a function of frequency for different concentration of WHF in EPDM and (b) the ac conductivity and binding energy as a function of the WHF concentration. The error bars are within the symbol’s sizes.

The influence of the electric field, which is reflected by an increase in frequency, is shown to improve electrical conductivity in semiconductors and, in a manner comparable to that, in polymers^77^;

23 $${\sigma _{ac}}\left( \omega \right)=A{\omega ^s}.$$

where *A* is a constant, $\:\omega\:$ is the angular frequency, *s*-parameter represents the factor which gives an indication on the conduction mechanism^78^. The relationship between ac conductivity and angular frequency is depicted in Fig. 8a, where it is clear that $\:Ln\left[{\sigma\:}_{ac}\left(\omega\:\right)\:\right(S/m\left)\right]$ increases with frequency, precisely matching Eq. (17). It is commonly found that the presence of WHF increases the electrical conductivity of the EPDM samples regarding the effect of WHF ratios on conductivity, as shown in Fig. 8b. At a 30 phr concentration of WHF, the conductivity increased, and then it was reduced at a 40 phr concentration of WHF and then returned to increase again at a 50 phr concentration of WHF. These results are in exact agreement with the obtained results from the free volume contents (*I*_*3*_) versus WHF concentration [Fig. 6b].

Two mechanisms are known for the conduction process: quantum mechanical tunnelling (QMT) and correlated barrier hopping (CBH) conduction mechanisms. The QMT is a phenomenon where electrons can cross a potential barrier while lacking the necessary energy to do so. The ability of electrons to tunnel past obstacles and behave like waves makes this possible. In this mechanism, as the electrons approach the barrier, they run across an energy gap that they are unable to bridge. There is a limited chance that they will be able to burrow through the barrier and carry on their course, though, because of their wave-like nature. For the QMT mechanism, *s*-parameter is independent of temperature and is given as^79^;

24 $$s=1 - \frac{4}{{ln\left( {\frac{1}{{\omega \tau }}} \right)}}.$$

where $\:\tau\:$ is the relaxation time of the charge carriers. On the other hand, the CBH conduction mechanism is used to explain the behaviour of charge carriers in disordered semiconductors and organic materials. In these materials, the charge carriers can become trapped in localized states due to disorder and defects. To move through the material, the charge carriers must hop from one localized state to another by overcoming energy barriers. In such a process, the particles move by hopping over energy barriers that are correlated with each other, meaning that the height and position of each barrier are influenced by the presence of neighbouring barriers. For the CBH mechanism, the *s*-parameter can be calculated by the relation^80,81^;

25 $$s=1 - \frac{{6kT}}{{{W_m}+kT \cdot ln\left( {\omega \tau } \right)}}.$$

where $\:{W}_{m}$ represents the binding energy that the charge carriers need to hop over the barrier. *T* is the absolute temperature, and *k* is the Boltzmann constant. According to 1st approximation, $\:{W}_{m}$ is so greater than the term $\:kT\bullet\:Ln\left(\omega\:\tau\:\right)$, then it leads to:

26 $$s=1 - \frac{{6kT}}{{{W_m}}}.$$

The *s*-parameter values for different WHF concentrations have been calculated from the slope of the relation between Ln [σ_ac_] and Ln (ω) as listed in Table 5. It is less than unity, indicating that the primary conduction mechanism is the correlated barrier hopping process. Table 5 also lists the calculated values of barrier height *W*_*m*_ for EPDM with different concentrations of WHF. As seen in Fig. 8b, the values of the barrier height versus the WHF concentration demonstrate the opposite behaviour of the conductivity, where the higher the binding energy or barrier height, the lower the conductivity.

**Table 5:** Variation of s-parameter and barrier height with WHF concentration.

WHF concentration (phr)	s-parameter	W_m_ (eV)
0	$0.93 \pm 0.006$	$2.22 \pm 0.16$
30	$0.89 \pm 0.004$	$1.41 \pm 0.05$
40	$0.94 \pm 0.007$	$2.59 \pm 0.30$
50	$0.81 \pm 0.006$	$0.82 \pm 0.03$

**Fig. 9:**
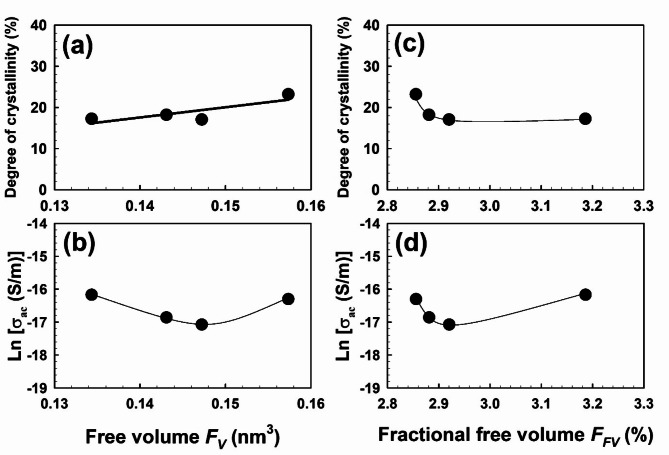
Free volume and fractional free volume dependence of the degree of crystallinity and electric conductivity. The error bars are within the symbol’s sizes.

In general, a pure polymer’s free volume and mechanical properties depend on its crystallinity, any dipole–dipole interactions that may exist, hydrogen bonds, and internal molecular entanglements. On the other hand, in the composite instance, other elements come into play, including the glass transition, crosslink density, interfacial interaction, and the polymer chains’ limited mobility. The relationship between macroscopic and nanostructure characteristics is shown in Fig. 9 for EPDM-WHF Fibre composites, where the free volume is drawn as a function of crystallinity [Fig. 9a] and electrical conductivity [Fig. 9b]. On the other hand, the fraction of free volume or the content of free volume is drawn as a function of crystallinity [Fig. 9c] and electrical conductivity [Fig. 9d]. From these figures, it is found that free volume controls the macroscopic properties of the polymer. The figure illustrates how WHF significantly affects the composite structure, whereby an increase in WHF concentration results in a drop in free volume due to an increase in crosslink property. Consequently, the dielectric constant of the EPDM-WHF fibre composites will decrease. While the free volume content increased as WHF increased, the composites’ ac conductivity increased dramatically as WHF increased despite the free volume content decreasing. The conductivity of the polymer and the amount of free volume in the polymer matrix are correlated linearly in some regions. In our instance, the dominating crosslinking feature caused the free volumes to decrease as the WHF concentration increased. As the free volume increases within the range of 0.13 to 0.16 nm³, the ac conductivity shows a slight decline, particularly dipping around Fv ≈ 0.14 nm³. This suggests that the increased free volume, which typically facilitates the mobility of charge carriers and could enhance conductivity, does not significantly improve the ac conductivity in this composite material under investigation. Instead, the observed decrease in conductivity may be linked to the effects of WHF concentration; as WHF concentration rises, the free volume decreases due to enhanced crosslinking properties. This increase in crosslinking likely restricts the movement of charge carriers, counteracting the potential benefits of the increased values of free volume.

## Conclusion

The decrease in swelling degree and the increase in crosslinking density values as a function of EPDM loading WHF were explained by the fact that when the WHF grew, the chain entanglements also increased, which reduced the solvent uptake. Due to the WHF fibres’ ability to stick together in bundles and to withstand the dispersion of individual fibres as fibre content increases-that is, the lack of adhesion between the fibres and rubber matrix, i.e. the tensile qualities of the EPDM-WHF vulcanizates exhibit poor tensile strength. The incorporation of WHF into EPDM resulted in an enhancement of their ability to store electrical energy at lower frequencies. The samples exhibited a broad peak in their dielectric loss, indicating a relaxation behavior associated with the presence of free volume in the material. Moreover, the addition of WHF generally led to an improvement in the electrical conductivity, with the highest conductivity observed when the WHF concentration was at 30 phr of WHF. The dominant conduction mechanism in the composites was analyzed, and it was found that the CBH mechanism played a major role. The values of the s-parameter suggest the existence of localized states and energy barriers. The free volume content of the composites played a crucial role in determining their overall properties. As the concentration of WHF increased, the free volume decreased due to increased crosslinking, resulting in an increase in the dielectric constant. However, despite the decrease in free volume, the ac conductivity showed a significant increase as the WHF concentration increased. This indicates that the higher concentration of free charge carriers contributed to the enhanced conductivity through the CBH mechanism.

## Data Availability

The datasets used and/or analysed during the current study are available from the corresponding author on reasonable request.
